# Acute sporadic hepatitis E in the Zhejiang coastal area of China: a 14-year hospital-based surveillance study

**DOI:** 10.1186/s12985-019-1119-7

**Published:** 2019-02-04

**Authors:** Jun Tan, Yijuan Chen, Lin Wang, Ta-Chien Chan, Said Amer, Xiaobin Xu, Jian Cai, Wei Li, Xiaoqing Zheng, Mi Zhou, Shuwen Qin, Na Zhao, Ziping Miao, Shelan Liu

**Affiliations:** 1 Department of Hepatology, Ningbo No.2 Hospital, Ningbo, Zhejiang Province China; 2 Department of Infectious Diseases, Zhejiang Provincial Center for Disease Control and Prevention, 3399 Binsheng Road, Binjiang District, Hangzhou, (310051) Zhejiang Province China; 3 Department of Microbiology and Infectious Disease Center, School of Basic Medical Sciences, Peking University Health Science Center, Beijing, China; 4 Research Center for Humanities and Social Sciences, Academia Sinica, Taipei, Taiwan; 5 Department of Zoology, Faculty of Science, Kafr El Sheikh University, Kafr El Sheikh, Egypt; 6 School of Automation, Hangzhou Dianzi University, Hangzhou, China; 7 The Medical School of Ningbo University, Ningbo, Zhejiang Province China; 8 CAS Key Laboratory of Pathogenic Microbiology and Immunology, Institute of Microbiology, Chinese Academy of Sciences, Beijing, China

**Keywords:** Hepatitis E, Epidemiology, Trends, High risks, Coastal areas

## Abstract

**Background:**

To examine the epidemiological trends and changes of hepatitis E virus (HEV) infection and the potential risk factors for severe infection in the Zhejiang eastern coastal area of China.

**Methods:**

We analyzed statutory hepatitis E cases notifications and inpatient data held by the national surveillance and hospital information systems in Wenzhou, Taizhou, Ningbo, and Zhoushan cities of the Zhejiang eastern coastal area of China.

**Results:**

Nine thousand four hundred sixteen hepatitis E cases were reported from 2004 to 2017, with an average incidence of 2.94 per 100,000. The overall death rate was 0.06% (6/9416). A gradual decline of hepatitis E cases was found in the coastal areas since 2007, while a rise was identified in the non-coastal areas. Annual incidence in non-coastal cities was much higher than that in coastal cities (4.345 vs. 2.945 per 100,000, relative risk = 1.5, *P* value < 0.001). The mean age was 52 years old and 50.55 years with a male-to-female ratio of 2.32:1 and 2.21:1 in coastal and noncoastal areas respectively (all *P* > 0.05). Hepatitis E cases prevalence increased with age, highest among men in their 70s (9.02 vs. 11.33 per 100,000) and women in their 60s (3.94 vs. 4.66 per 100,000) groups for both coastal and noncoastal areas respectively. A clear seasonal pattern was observed, with a peak in March (0.4429 per 100,000) in coastal areas. 202 inpatients were documented, of which 50.50% (102/202) were severe cases. Male individuals with alcohol consumption, alcohol hepatic diseases, and superinfection were the three independent highest risks for severe infections (all with *P* value < 0.05).

**Conclusions:**

This is to our knowledge the largest epidemiological study of hepatitis E cases in the eastern coastal area of Zhejiang province of China. The patterns of infection across the coastal areas were similar to those of the non-coastal areas, but the incidence was substantially lower and decreased gradually since 2007.

## Background

Hepatitis E virus (HEV) is a non-enveloped positive-sense RNA virus and the only member of the family *Hepeviridae* [1]. HEV strains frequently related to human infections belong to species *Orthohepevirus* A, in which there are 8 major genotypes (HEV1–8) [2]. To date, five genotypes of mammalian HEV (genotypes 1–4 and 7) are known to affect humans [2, 3]. HEV1 and 2 only infect humans, and are responsible for waterborne epidemics in developing regions [3], whereas zoonotic genotypes HEV3, HEV4 and HEV7 might infect human beings via foodborne transmission from different animal reservoirs (pigs and camels), but the principal reservoir is domestic pigs (HEV3 and 4), three genotypes are responsible for sporadic cases in both developed and developing countries [3–5].

Hepatitis E is predominantly circulating in many developing countries in Asia, Africa, and Latin America [6, 7], especially in countries with pig farms and coastal areas [8]. China is recognized as a hyperendemic area for hepatitis E [9, 10], where a high seroprevalence has been observed among the overall population (23.46%) [11]. The epidemiological and etiological changes have been gradually identified in China since the largest HEV outbreak in 1986 in Xinjiang Uighur autonomous region of China [12]. First of all, the predominant HEV genotype in China has changed from genotype 1 to genotype 4 [13]. Secondly, the number of hepatitis E cases increased, with an annual percentage change of 7% in China overall, with eastern China having the highest prevalence [14]. Thirdly, a higher health risk of hepatitis E cases was found in the workers who have direct contact with raw seafood or users of water and consumers of shellfish contaminated by HEV in the Bohai coastal area of China [15, 16]. A series of articles have recorded hepatitis E incidence in the inlands of China, especially in rural areas [9, 10]. However, human beings infected with HEV in the coastal area of eastern China appear to be extremely limited.

This study aimed to elucidate the epidemical trends and changes as well as the related risk factors among east-coast residents in Zhejiang Province, China. We reviewed China’s National Notifiable Disease Report System and hospital information systems (HIS) in four coastal cities (Wenzhou, Taizhou, Ningbo, and Zhoushan cities, Zhejiang Province) along the East China Sea, with 24,287,966 Chinese residents, accounting for 43.45% of the general population (55,899,795) in Zhejiang Province, 2017. This location is one of the most densely populated urban agglomeration areas in Zhejiang. The information extracted from this study is needed to better understand the epidemiological profile of hepatitis E cases, and will be valuable for the extensive administration of the HEV vaccine in coastal areas of China in the future. To our knowledge, this is the largest epidemiological study that has been done on hepatitis E cases in coastal areas of Zhejiang Province.

## Materials and methods

### Case definitions

An acute viral hepatitis (AVH) case was defined as an individual having an acute illness with a discrete onset of any sign or symptom (e.g., fever, headache, malaise, anorexia, nausea, vomiting, diarrhea, or abdominal pain) and either jaundice or elevated serum alanine aminotransferase levels higher than 100 IU (United States Centers for Disease Control and Prevention, 2012). We defined hepatitis E cases based on the date of disease onset and on the updated diagnostic criteria issued by the Chinese Ministry of Health in 2008; these criteria are based on epidemiologic history, clinical signs, and laboratory test results [17]. Mild, moderate, and severe infections were defined according to the Child–Pugh score for grading the severity of liver disease [18, 19].

### Data source

We reviewed the sporadic cases of hepatitis E reported during from 2004 to 2017 from China’s National Notifiable Disease Report System. We extracted the epidemic data of 9416 confirmed hepatitis E cases in four coastal cities (Taizhou, Wenzhou, Ningbo and Zhoushan) and 17,968 confirmed hepatitis E cases in seven non-coastal cities(Hangzhou, Jinhua, Quzhou, Jiaxing, Shaoxing, Lishui, and Huzhou), including the age, gender, occupation, onset date and month, outcome. The demographic information is from the China National Bureau of Statistics (with data updated by the end of each year).

A total of 202 admitted cases were included in this retrospective study during this period. All were confirmed and come randomly from the 9416 cases from coastal areas during 2004 to 2017. We also collected the following information from these inpatients from HIS in four coastal cities: age, sex, diagnosis, medical history, clinical symptoms, and biochemical indicators. The hospitals are territorial referral centers for acute hepatitis diagnosis and treatment in local cities.

### Laboratory testing

All serum samples were tested for anti-hepatitis A virus (HAV), hepatitis B virus (HBV) surface antigen, and anti-hepatitis C virus (HCV) antibodies along with anti-HEV IgG and IgM assays for the diagnosis of acute hepatitis E. All reagents for these assays were supplied by Abbott Laboratories (IL, USA) except IgM anti-HEV, which was supplied by Wan Tai Pharmaceutical Co. (Beijing, China). Routine blood investigations, including complete blood picture, coagulation profile, and liver function tests, were performed upon admission. The peak values during the time of admission to the hospital were collected.

### Statistical analysis

The continuous variables are expressed as mean ± SD. The days from clinical onset to be diagnosed, hospitalized and discharged are expressed as median ± SD because they are non-normally distributed. The categorical variables are presented as the number (%).T test and Non-parametric test was used for the continuous variables. The Chi-square test or Fisher’s exact test was used to analyze the categorical variables and to assess an odds ratio(OR). To further confirm the risk factors for severe liver diseases, a multivariate logistic analysis was used for examining the variables which were found to be significant in univariate analyses or be suspected as potential risk factors from published articles [20]. All Statistical analyses were performed using SPSS 19.0 software (SPSS Inc., Chicago, IL). The statistical tests were two-sided, and significance was defined as *P* < 0.05.

## Result

### Epidemiological characteristics

**Fig. 1:**
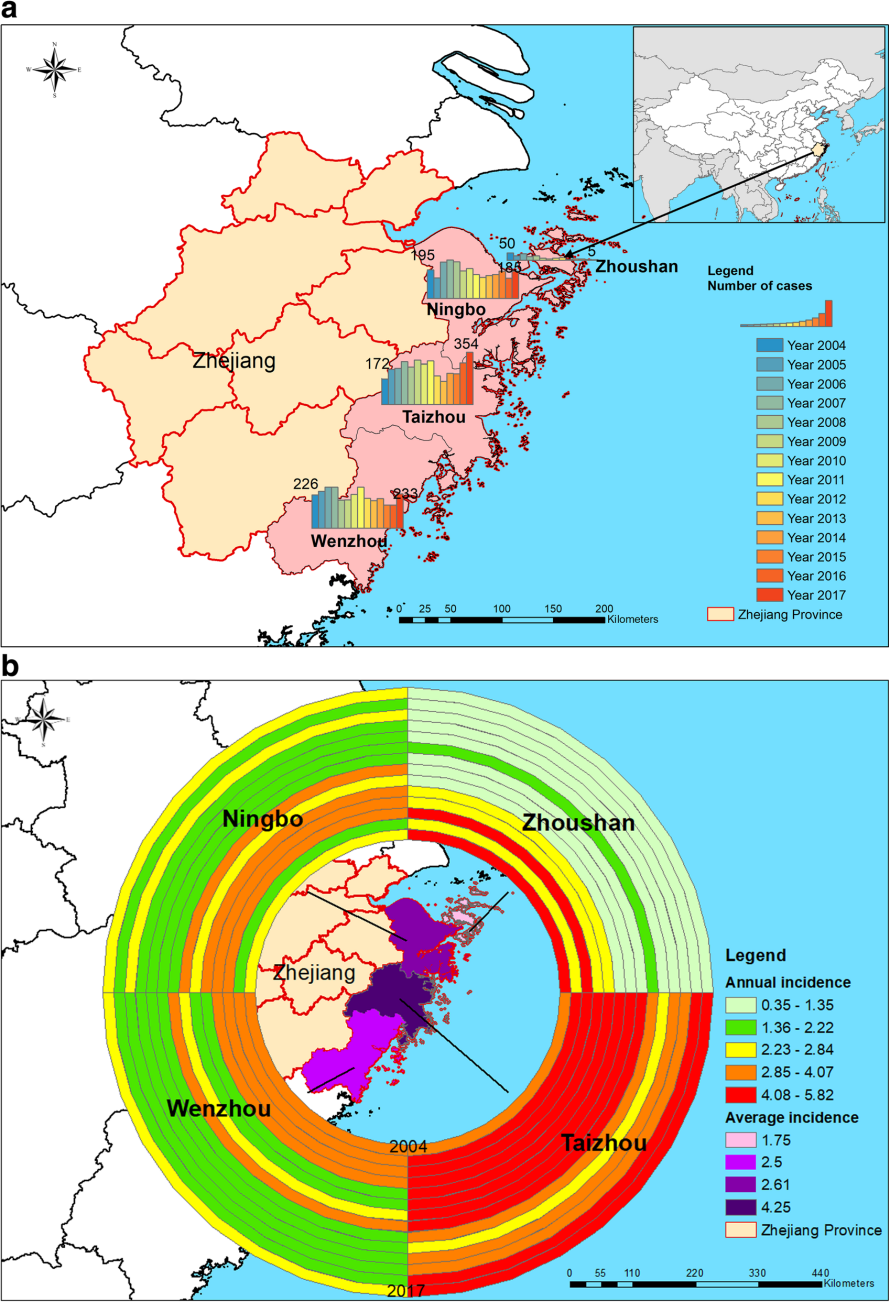
Geographical distribution of human hepatitis E cases reported in east coastal areas of Zhejiang Province, China (*N* = 9416), 2004–2017. **a**: Number of cases; **b**: annual incidence and the average incidence

**Fig. 2:**
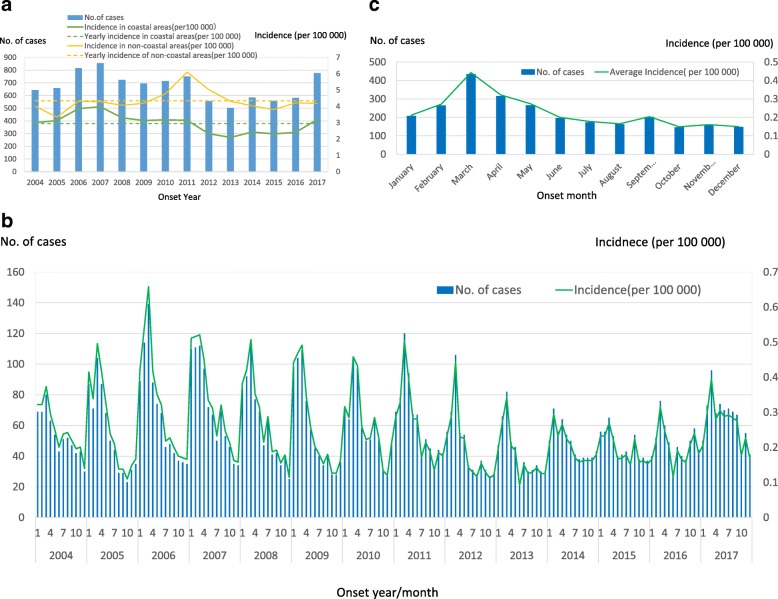
Incidence and number of human hepatitis E cases reported in east coastal areas (*N* = 9416) and non-coastal areas of Zhejiang Province, China (*N* = 17,968), 2004–2017. Notes: **a**: Yearly incidence; **b**: Yearly and month incidence; **c**: Monthly average incidence

From 2004 to 2017, a total of 9416 human acute hepatitis E cases were reported in the four east-coast areas of Zhejiang, accounting for 2.14–7.13% of acute hepatitis, with a case–fatality rate of 0.06% (6/9416). Figure 1a showed that the changes of the number of HEV cases in four coastal cities. The map indicated the annual cases decreased in the past 14 years except Taizhou, increased from 172 cases in 2004 to 354 cases in 2017. Figure 1b suggested the geographical distribution of HEV incidence in four cities, which Taizhou was the most affected area. The annual average cases were 673 during 2004–2017 and the annual incidence averaged 2.94 cases per 100,000 individuals from 2004 to 2017. Since starting the disease surveillance in 2004, the incidence of hepatitis E cases in coastal areas increased slightly and peaked in 2007, with an occurrence rate of 3.98 per 100,000. Thereafter, the incidence rate gradually decreased to the lowest rate in 2013, with an incidence of 2.10 per 100,000. The incidence started to slowly increase again starting in 2014 (Fig. 2a). By contrast, the reported rate in non-coastal areas increased quickly and reached a peak in 2011, with an incidence of 6.10 per 100,000, sharply decreased to the lowest in 2015, with an incidence of 3.80 per 100,000. The yearly hepatitis E cases incidence in non-coastal cities was much higher than that in coastal cities (4.345 per 100,000 vs. 2.945 per 100,000, relative risk = 1.5, *P* value < 0.001) (Fig. 2a).

Generally, the seasonal distribution of this pattern did not vary in different years (Fig. 2b). Cases of acute hepatitis E occurred throughout the year. However, the number of hepatitis E cases in January to May accounted for 47.29% (4453/9416) of the total cases from 2004 to 2017. Only one single peak occurred in the eastern coastal area of Zhejiang every year (Fig. 2b). As for monthly distribution, it peaked annually in March [accounting for 14.43% (1359/9416) of total cases], and the lowest incidence was identified in October [accounting for 5.39% (507/9416)] (Fig. 2c).

**Fig. 3:**
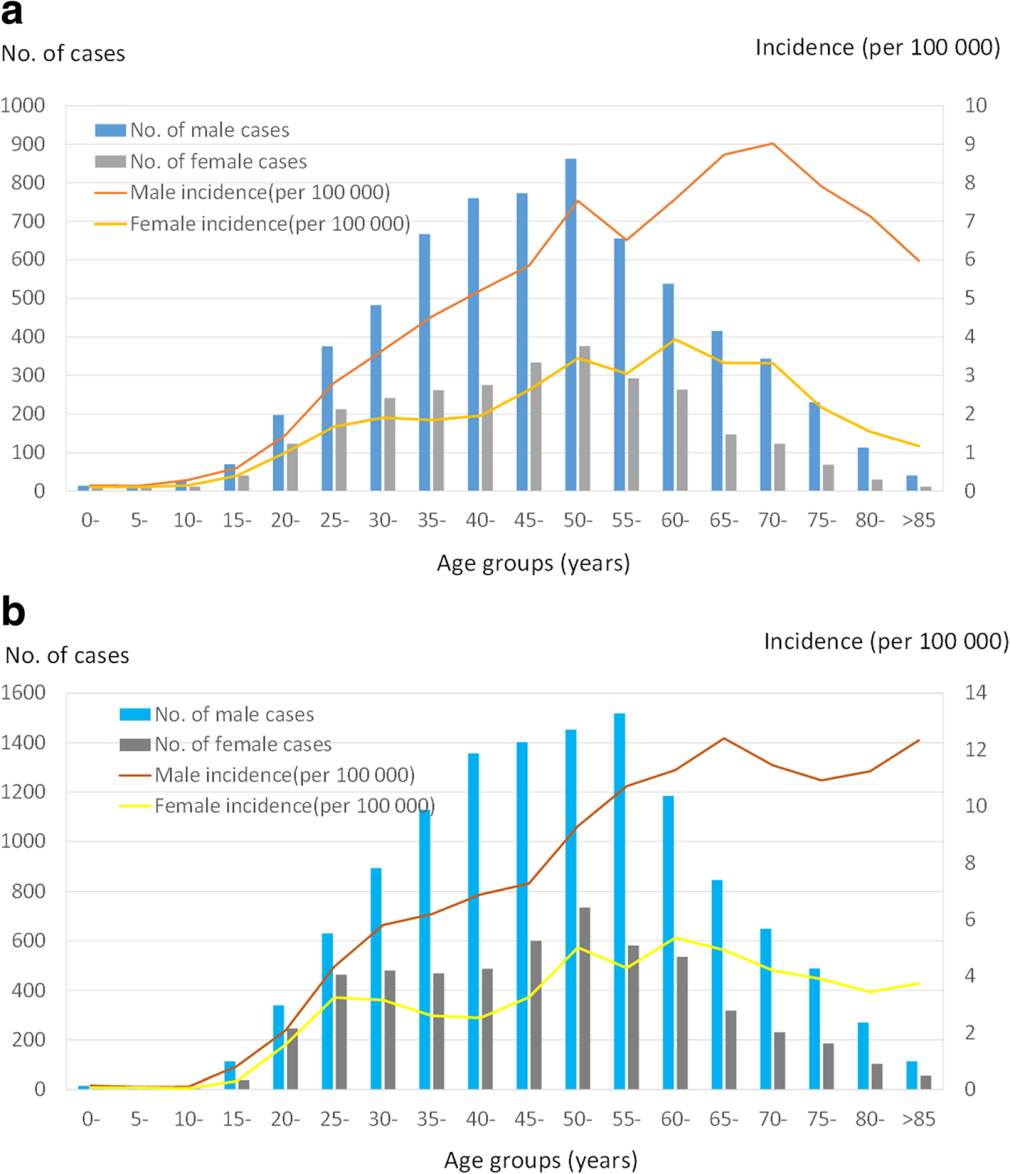
Overall yearly incidence of hepatitis E cases by sex and age reported from east coastal and non-coastal areas of Zhejiang Province, China, 2004–2017. **a**: coastal areas (*N* = 9416); **b**: non-coastal areas (*N* = 17,968)

The mean age of the reported cases was 52 years (range: 11 months–94 years) and 50.55 years (range: 8 months–92 years) in coastal areas and non-coastal areas respectively (*P* = 0.078). A high incidence rate was observed in the age category of 40–59 years, accounting for 45.99% (4330/9416) and 45.26% (8132/17968) of the total reported cases in coastal and non-coastal areas respectively (Fig. 3a, b). By contrast, a low incidence rate was found in children younger than 15 years old (0.89%, 84/9416) and 0.29% (54/17968) in coastal and non-coastal areas respectively (Fig. 3a, b). The incidence increased with age in the groups aged greater than 15 years and reached the highest point among men in their 70s (9.02 per 100,000) and women in their 60s (3.94 per 100,000) (Fig. 3a). The same results were seen in noncoastal areas (Fig. 3b).

The overall annual incidence of hepatitis E cases was higher in males than in females across all age groups (2.32:1, *P* < 0.001) and (2.21:1, *P* < 0.001) in coastal and non-coastal areas respectively. In general, there was no difference in the gender distribution between coastal and noncoastal groups (*P* = 0.199).

In occupation distribution, farmers, workers, retirees, housekeepers and businessmen together accounted for 76.96% (7247/9416) and 81.72% (14,683/17968) of total cases between coastal and noncoastal areas respectively, with a significant difference between the two groups (*P* < 0.001) (Table 1). This occupational pattern was consistent year by year since 2004 in coastal and noncoastal cities.

**Table 1:** Occupational distribution of hepatitis E cases reported from east-coast and non-coastal areas of Zhejiang Province, China in 2004–2017

Occupations	Coastal areas (%, n)	Non-coastal areas (%, n)	*P* value
Farmers	44.36% (4177)	51.76% (9301)	*P* < 0.001
Housekeepers	10.05% (946)	4.04% (726)	
Workers	12.37% (1165)	11.20% (2012)	
Retirees	5.59% (526)	8.58% (1542)	
Others	6.44% (606)	1.39% (249)	
Officers	3.90% (367)	5.58% (1003)	
Businessmen	4.60% (433)	6.14% (1103)	
Migrant labor	2.51% (236)	2.83% (509)	
Students	1.67% (157)	0.90% (161)	
Teachers	1.26% (119)	1.34% (241)	
Food industry	0.98% (92)	0.86% (154)	
Fishermen	0.63% (59)	0.06% (10)	
Non-nursery governess	0.27% (25)	0.04% (8)	
Medical workers	0.52% (49)	0.65% (116)	
Public service	0.30% (28)	0.21% (38)	
Seafarers and long-distance drivers	0.12% (11)	0.10% (18)	
Nursery governess	0.04% (4)	0.02% (3)	
Herdsman	0.03% (3)	0.04% (7)	
Nursed baby	0.05% (5)	0.13% (24)	
Unknown	4.31% (406)	4.14% (743)	
Total	100% (9416)	100% (17,968)	

Notes: We used the chi-square test to compare the occupational distribution between coastal and non-coastal areas of Zhejiang Province, China during 2004–2017

### General information for HEV admitted cases

During the study period, 202 hepatitis E confirmed inpatients were collected from the Department of Infectious Diseases or the local hepatitis reference center for diagnosis and treatment. Among these inpatients, mild, moderate and severe cases accounted for 28.22% (57/202), 21.28% (43/202), and 50.50% (102/202) of all inpatients respectively. The mean age was 52 years among these admitted patients, and male cases accounted for 79.21% (160/202).

44.06% (89/202) of these inpatients were identified as having chronic liver diseases, with underlying infection with another hepatitis virus [30.69% (62/202)], alcoholic liver disease [14.36% (29/202)], and liver cirrhosis [6.93% (14/202)] serving as important acute precipitants leading to hepatitis E cases’ admission. The distribution of the various hepatitis etiological agents among these 62 hepatitis E inpatients was as follows: 1.61% (1/62) HAV, 96.77% (60/62) HBV, and 1.61% (1/62) HCV

### Clinical manifestation for hepatitis E inpatients

The clinical presentations were as follows: Fatigue was the most frequent symptom (188 patients, 93.07%), followed by dark urine (182 patients, 90.10%). Jaundice was the most predominant physical examination [84.65% (171/202)]. The alanine aminotransferase (ALT) activities varied at 42–10,038 IU/L, with a peak level of 1744 IU/L. The aspartate aminotransferase (AST) activities peaked at 1453 IU/L (range: 56–8415 IU/L) and direct bilirubin at 109 (range: 3.6–523.4) μmol/L. The mean prothrombin activity (PT) and albumin (ALB) at the peak level were 15 s and 34 g/dL, respectively in those patients.

### The high risks of acute severe HEV infections

We compared the epidemic features between severe and non-severe groups to find out the high risks related to the severe and super infections. The results showed that the risk leading to severe infections sharply increased in male patients, in whom alcohol consumption, frequent smoking, and alcoholic liver disease pre-existed before the time of hepatitis E onset (all *P* value< 0.05, Table 2). We further identified that the superinfection with HEV was triggered by hepatic medication and liver cirrhosis (Table 3).

**Table 2:** Assessment of high risks associated with disease severity among 202 acute sporadic HEV inpatients from the east- coast area of Zhejiang Province, China, 2004–2017

Valuable	Non-severe cases (*n* = 100)	Severe cases (*n* = 102)	OR (95% CI)	*P* value
Demographic characteristics				
Age range (years)	23–80	24–83	/	
Mean age ± SD (years)	50.63 ± 12.51	51.91 ± 13.81	/	0.49^d^
Sex ratio (male/female)	2.33 (70/30)	7.50 (90/12)	0.311 (0.149–0.651)	< 0.01^a^
Educational level (<college) (%, n)	87.00 (87/100)	92.16 (94/102)	0.570 (0.225–1.440)	0.23^a^
Smoker (%, n)	20.00% (20/100)	41.18% (42/102)	2.800 (1.493–5.252)	< 0.01^a^
Alcohol user (%, n)	28.00% (28/100)	49.02% (50/102)	2.473 (1.379–4.434)	< 0.01^a^
History of blood transfusion (%, n)	4.00% (4/100)	4.90% (5/102)	1.237 (0.322–4.747)	1.00^b^
Hepatoxic medication (%, n)	11.00% (11/100)	13.73% (14/102)	1.287 (0.554–2.990)	0.56^a^
Chronic liver diseases (%, n)	36.00% (36/100)	51.96% (53/102)	1.923 (1.094–3.378)	0.02^a^
Hepatitis A	1.00% (1/100)	0 (0/102)	0.990 (0.971–1.010)	0.50^b^
Hepatitis B	24.00% (24/100)	35.29% (36/102)	1.727 (0.936–3.188)	0.08^a^
Hepatitis C	1.00% (1/100)	0 (0/102)	0.990 (0.971–1.010)	0.50^b^
Liver cirrhosis	4.00% (4/100)	9.80% (10/102)	2.609 (0.790–8.612)	0.10^a^
Fatty liver disease	4.00% (4/100)	7.84% (8/102)	2.043 (0.595–7.012)	0.25^a^
Alcoholic liver disease	9.00% (9/100)	19.61% (20/102)	2.466 (1.063–5.720)	0.03^a^
Hepatic carcinoma	1.00% (1/100)	2.94% (3/102)	3.000 (0.307–29.337)	0.62^b^
Drug-induced hepatitis	1.00% (1/100)	0.98% (1/102)	0.980 (0.060–15.889)	1.00^b^
Chronic nonliver comorbidities (%, n)			/	0.75^c^
No comorbidities	56.00% (56/100)	60.78% (62/102)	/	
1 comorbidity	27.00% (27/100)	23.53% (24/102)	/	
2 comorbidities	12.00% (12/100)	8.82% (9/102)	/	
≥ 3 comorbidities	5.00% (5/100)	6.86% (7/102)	/	
Disease progression				
Median days from onset to consultation	6 (0–39)	6 (0–24)	/	0.93^c^
Median days from onset to admission	7 (1–49)	7 (2–38)	/	0.12^c^
Median days from onset to confirmation	11.5 (4–54)	11 (4–43)	/	0.75^c^
Median days from onset to discharge	27 (9–71)	36.5 (14–111)	/	< 0.001^c^
Days hospitalized	18 (5–46)	26 (7–108)	/	< 0.001^c^

Notes: Non-severe cases denote mild and moderate cases; *SD* standard deviation, ^a^ = Chi-square test; ^b^ = Fisher test; ^c^ = Non-parametric test; and ^d^ = T test in *P* value column; “/” denotes not available

**Table 3:** Assessment of high risks associated with super HEV infection among 202 acute sporadic HEV inpatients from east-coast area, Zhejiang, China, 2004–2017

Valuable	Single HEV infection (*n* = 141)	Super HEV infection (*n* = 61)	OR (95% CI)	*P* value
Demographic characteristics				
Age range (years)	23–83	26–82	/	
Mean age ± SD (years)	52.42 ± 13.19	48.64 ± 12.84	/	0.06^d^
Sex ratio (male/female)	3.55 (110/31)	4.55 (50/11)	0.781 (0.363–1.677)	0.53^a^
Smoker (%, n)	30.50% (43/141)	31.15 (19/61)	1.031 (0.538–1.974)	0.93^a^
Alcohol user (%, n)	38.30% (54/141)	39.34% (24/61)	1.045 (0.565–1.934)	0.89^a^
History of blood transfusion (%, n)	3.55% (5/141)	6.56% (4/61)	1.909 (0.495–7.368)	0.46^b^
Hepatoxic medication (%, n)	4.26% (6/141)	31.15% (19/61)	10.179 (3.816–27.147)	< 0.001^a^
Chronic liver diseases (%, n)	19.15% (27/141)	40.98% (25/61)	2.932 (1.515–5.767)	< 0.001^a^
Liver cirrhosis	2.13% (3/141)	18.03% (11/61)	10.120 (2.712–37.768)	< 0.001^b^
Fatty liver disease	5.67% (8/141)	6.56% (4/61)	1.167 (0.338–4.030)	0.76^b^
Alcoholic liver disease	12.06% (17/141)	19.67% (12/61)	1.786 (0.795–4.014)	0.16^a^
Hepatic carcinoma	0.71% (1/141)	4.92% (3/61)	7.241 (0.738–72.064)	0.08^b^
Drug-induced hepatitis	1.42% (2/141)	0% (0/61)	/	1.00^b^
Chronic nonliver comorbidities (%, n)			/	0.87^c^
No comorbidities	56.74% (80/141)	62.30% (38/61)	/	
1 comorbidity	26.24% (37/141)	22.95% (14/61)	/	
2 comorbidities	11.35% (16/141)	8.20% (5/61)	/	
≥ 3 comorbidities	5.67% (8/141)	6.56% (4/61)	/	
Disease progression				
Median days from onset to consultation	6 (0–39)	5 (0–24)	/	0.06^c^
Median days from onset to admission	7 (2–40)	7 (1–49)	/	0.51^c^
Median days from onset to confirmation	11 (4–45)	11 (4–54)	/	0.83^c^
Median days from onset to deterioration	9 (0–32)	7 (0–35)	/	0.56^c^
Median days from onset to discharge	29 (9–111)	33 (10–73)	/	0.11^c^
Days hospitalized	21 (5–108)	24 (6–64)	/	0.15^c^

Notes: *SD* standard deviation; ^a^ = Chi-square test; ^b^ = Fisher test; ^c^ = Non-parametric test; and ^d^ = T test in *P* value column; “/” denotes not available

Application of multivariate logistic regression models indicated that alcohol consumption was the only variable significantly affecting the severity of the disease (Table 4). The model estimated that odds of disease severity were mildly higher for persons with consumed alcohol than for others [odds ratio, 0.5215, 95% CI (0.2574~1.0576), *P* = 0.07]. No other covariates were significant (Table 4).

**Table 4:** Multivariate logistic regression model assessing odds ratios of risk associated with severity of 202 cases caused by hepatitis E virus, from eastern coastal areas of Zhejiang Province, China, 2004–2017

Variable	Value	Odds ratio (95% CI)	*P* value
Mean Age (Years)	52 (11 months~ 94 years)	0.9990 (0.9734~1.0243)	0.925
Gender ratio (Male/Female)	2.59	1.4623 (0.7033~3.0374)	0.309
Smoker (%,n)	30.69% (62/202)	0.6325 (0.3042~1.3152)	0.22
Alcohol user (%,n)	38.61% (78/202)	0.5215 (0.2574~1.0576)	0.071
Rural/ Urban ratio	1.5 (120/80)	1.3526 (0.6798 ~ 2.6912)	0.389
Chronic liver diseases, no. (%)	25.74% (52/202)	0.7741 (0.5505~1.0876)	0.14
Hepatic medicine (%,n)	12.38% (25/202)	0.9176 (0.3599~ 2.3420)	0.858
Median days from onset to diagnosis	11 (0~40)	1.0101 (0.9724~1.0492)	0.618
Super infection no. (%)	30.20% (61/202)	0.5374 (0.2704~1.0672)	0.076
Illiteracy no. (%)	5.45% (11/202)	0.5644 (0.0902~3.5325)	0.541

### Clinical process and outcome

No differences were found between severe and non-severe cases in the median days from onset to consultation, onset to hospitalization, and onset to confirmation. However, the median days from onset to being discharged [36.5 days vs. 27 days, *P* < 0.001] and hospital stay [26 days vs. 18 days, *P* < 0.001] were much longer in the severe cases than in the non-severe cases (Table 2). No deaths were reported in these 202 inpatients.

## Discussion

On the basis of a 14-year period of hospital surveillance, we described an ongoing decreasing trend of acute sporadic hepatitis E in the east-coast area of Zhejiang. Through more than 9000 confirmed hepatitis E cases, this largest study discovered the epidemiological trend and changes as well as high risks for hepatitis E cases who lived in the developed coastal areas of Zhejiang.

This study demonstrated that the number of cases in the east-coast area of Zhejiang showed a decreasing trend since 2007. In coastal areas where swine are raised, swine manure could be a source of HEV contamination of irrigation water or coastal waters with concomitant contamination of produce or shellfish [15, 21]. The consumption of this contaminated seafood and water may contribute to the increasing risk of zoonotic foodborne HEV circulation [15, 16]. Our finding was in stark contrast to the experiences in the other parts of China and European countries [22–24]. For example, Jiang et al. have reported that the morbidity caused by HEV has been increasing over the recent decade and is centered on the coastal region of Yaitai of eastern China [23]. Mansuy et al. reported that the incidence of hepatitis E in southwest France was stable from 2003 to 2007 [24]. There may be several reasons for the decrease found in Zhejiang. First, the decrease in hepatitis E incidence could have resulted either from a true decrease in the number of cases or from the dominant genotype shift in China from G1 to G4 since 2000 [17, 25]. In coastal areas, seafood consumption increased from 4.15 million tons in 2004 to 4.91 million in 2015 in these areas [2016 Zhejiang Province of China Statistical Yearbook,], greater than the increase in pork consumption during that time; note that domestic pigs serve as an important reservoir for genotypes 3 and 4 [26, 27]. Second, the water supply and sanitation systems have been improved through projects to improve drinking water and lavatories by providing water pipe networks and water purification facilities as well as sewage treatment in rural areas of Zhejiang Province in 2009 (internal data from Zhejiang Centers for Disease Control and Prevention). These steps may have decreased the transmission and infection of HEV in these areas. Third, the decrease in HEV was related to stricter regulations on large-scale swine farming and centralized slaughter in recent years, including restricting release of pollutants such as feces and sewage to the external environment, occupational protection, and HEV surveillance.

The epidemiological analysis indicated no change in the seasonal pattern or demographic features of hepatitis E cases in the past 14 years [14, 17]. Our study showed that cases of acute hepatitis E occurred regularly throughout the year during the study period, but the incidence rate was highest during March. The annual pattern was similar to those of the inland cities of China [6, 17, 28]. However, the seasonality pattern remained distinct from those in northern India and Uganda [29]. The peak time may be associated with the pattern of food preparation and excessive meat and seafood consumption that occurs during this season, which coincides with the Chinese New Year [6, 13].

The age and gender profile of infection reported in these coastal areas is in agreement with that in previous studies: Men and farmers are at great risk of acute hepatitis E in the inlands of China and Europe, and most are middle aged or elderly [14, 17, 30, 31]. For patients aged over 50 years in particular, HEV infection increased with age. On one hand, these trends could be linked to the behavior of patients, occupational exposure, and/or broad social involvements in comparison with younger patients [13, 32]. On the other hand, HEV4 is the predominant strain among older patients in China, in comparison with epidemic HEV1 and HEV2, which predominate in young age groups [33]. These results are consistent with a study conducted in China’s coastal city of Yantai [23]. Conversely, the low incidence rates and the relative paucity of laboratory-confirmed cases in pediatric populations further strengthen the hypothesis that children are unrecognized or underdiagnosed because of anicteric hepatitis or subclinical infection [34, 35]. Asymptomatic infection is common with HEV3 and HEV4 [3, 33].

The clinical phenotypes of hepatitis E cases ranging from mild to fatal disease and extra-hepatic manifestation [33, 36] are not yet fully understood. Slightly over half of the patients (102 cases, 50.50%) in the current study were severe when admitted to hospital. We searched for the reasons for this disease severity, with a focus on host factors, and found that middle-aged men, especially smokers, had greater disease severity. Furthermore, alcohol consumers carried the highest risk for severe hepatitis E cases. The excessive alcohol consumption in coastal communities may play a role in the presentation of clinically overt hepatitis E infection [37]. Alcohol intake accelerates the degree of hepatic fibrosis, increases the risk of liver diseases, and worsens the clinical outcome of liver diseases [37–40]. Previous reports suggested an association between severe hepatitis infection and pre-existing diseases [41]. Indeed, 60.39% of the hepatitis E cases had pre-existing chronic liver diseases; among these cases, coinfection with hepatitis B virus and alcoholic liver disease were the most common. Altogether, older men who are high alcohol consumers, those with alcoholic liver disease, and HEV-superinfected individuals have a greater risk of developing severe diseases in the coastal areas of China relative to the other areas in China [13, 39].

We documented 202 cases of autochthonous hepatitis E infection in the eastern coastal area of Zhejiang. The number of hepatitis E cases accounted for approximately 4.4% of AVH. Affected patients in the coastal areas have hepatic symptoms similar to those seen in the inlands of China [20]. We found 84.65% of hepatitis E cases were jaundiced at presentation. The majority of severe hepatitis E infections had liver derangement, of which total bilirubin (TBi) and direct bilirubin (DBi) concentrations significantly increased compared to mild and moderate hepatitis E cases [21]. This finding indicates that these markers are the important predicators of severe hepatitis E infections.

This research found that the clinical progress of hepatitis E cases in the coastal areas of Zhejiang Province is very similar to that in the inland areas of China. The clinical progress and outcome are influenced by several factors, including the patient consultation time, diseases severity, medical diagnosis and treatment, etc. This study showed that the average days from illness onset to being discharged in the severe cases were much longer than in mild and moderate hepatitis E cases. The same result was found in the average days from admission to discharge from the hospital. This could be due to the longer antivirus treatment and restoration of liver functions for these hepatitis E cases [20]. At 4 weeks, all patient had normalized serum bilirubin or ALT levels and survived in these four eastern coastal areas of Zhejiang. The result is not consistent with the Monga et al. study, which found that superimposed acute hepatitis E infection with chronic liver disease has a protracted course (8 weeks) with high morbidity and mortality [42].

This study has several limitations. First, hepatitis E case surveillance was not thorough, and most cases (where the disorder was asymptomatic and mild, or the patient did not seek formal care) went undetected. Second, we did not analyze the changes in the diagnosis and report status in the past 14 years. Third, our study was based on the notifications of surveillance data, without examining the genotypes to estimate the shift of HEV genotypes in these coastal areas in Zhejiang.

A hepatitis E recombinant vaccine (HEV 239) was developed by Chinese scientists and licensed by the Chinese Food and Drug Administration FDA in 2012 [43, 44]. The broad application of HEV vaccination is the most efficient way to reduce hepatitis E cases in at-risk populations, as the HEV vaccine not only protects against hepatitis E infection but also decreases the severity [45]. These findings support the prioritization of HEV vaccination and the optimization of the timing of vaccination for those coastal residents of China in the future.

## Conclusion

This is the largest study to investigate the general epidemiology trend and the related risks factors of hepatitis E cases in the east-coast areas of Zhejiang Province, China. The results demonstrate a decrease in the incidence of hepatitis E cases since 2007 in the coastal areas, inconsistent with the increasing trend in the inlands of China. The incidence in coastal areas was much lower than the non-coastal areas. However, the seasonal and demographical features of hepatitis E cases in the coastal cities are similar to those non-coastal cities. Middle-aged men who are alcohol consumers, alcohol hepatic diseases, and superinfection are the three highest independent risk factors that induce severe hepatitis E infections in the eastern coastal areas of China.

## Abbreviations

ALB: Albumin
AVH: An acute viral hepatitis
DBi: Direct bilirubin
HAV: Hepatitis A virus
HBV: Hepatitis B virus
HCV: Hepatitis C virus
HEV: Hepatitis E virus
HIS: Hospital information systems
PT: Prothrombin activity
SD: Standard deviation
TBi: Total bilirubin
